# The AGM of Gauss, Ramanujan’s corresponding theory, and spectral bounds of self-adjoint operators

**DOI:** 10.1007/s00605-024-02051-0

**Published:** 2025-01-22

**Authors:** Markus Faulhuber, Anupam Gumber, Irina Shafkulovska

**Affiliations:** Faculty of Mathematics, University of Vienna, Oskar-Morgenstern-Platz 1, 1090 Vienna, Austria

**Keywords:** Arithmetic–geometric mean, Gabor system, Theta function, Lattice, Spectral bounds, Primary, 42C15, Secondary, 33C05, 33C67, 33C80

## Abstract

We study the spectral bounds of self-adjoint operators on the Hilbert space of square-integrable functions, arising from the representation theory of the Heisenberg group. Interestingly, starting either with the von Neumann lattice or the hexagonal lattice of density 2, the spectral bounds obey well-known arithmetic–geometric mean iterations. This follows from connections to Jacobi theta functions and Ramanujan’s corresponding theories. As a consequence, we rediscover that these operators resemble the identity operator as the density of the lattice grows. We also prove that the conjectural value of Landau’s constant is obtained as half the cubic arithmetic–geometric mean of $\root 3 \of {2}$ and 1, which we believe to be a new result.

## Introduction

In this work, we connect the problem of computing the spectral bounds of certain self-adjoint operators on $L^2(\mathbb {R} )$ with the arithmetic–geometric mean (AGM) of Gauss and Ramanujan’s corresponding theory. The operators involved arise from Gaussian Gabor systems over scaled von Neumann lattices and hexagonal lattices. To some extent, we can also draw conclusions for rectangular lattices. A common theme for all these lattices is that they contain certain root systems and, in fact, we cover all possible root lattices in dimension 2. Gauss has already shown that the squares of the classical Jacobi theta functions, which are used to define the elliptic modulus of complete elliptic integrals of the first kind, obey the arithmetic–geometric mean process. Moreover, it has been described by Mumford [57] how theta functions play an important role in the representation theory of the Heisenberg group. Interestingly, the specific spectral problem also arises from unitary representations of the Heisenberg group and is of importance in quantum mechanics and communication theory. To the best of our knowledge, a connection between the arithmetic–geometric mean iteration and the spectral problem has so far neither been observed nor been studied in the literature.

Restricting (the squares of) Jacobi’s theta functions to certain arguments allows us to connect to the spectral bounds for Gaussian Gabor frame operators over scaled von Neumann lattices, i.e., square lattices. We can then use a theory developed by Gauss to derive our first result. Moreover, Ramanujan’s corresponding theory for elliptic functions enters the scene and provides a cubic analog to the arithmetic–geometric mean. This has consequences for our considered spectral problem over the hexagonal lattice and gives our second result.

We denote the Gaussian function of $L^2(\mathbb {R} )$ unit norm by

$$\begin{aligned} \varphi (t) = 2^{1/4} e^{-\pi t^2}, \quad t \in \mathbb {R}, \ \left\Vert \varphi \right\Vert _2=1. \end{aligned}$$

For an element $(x,\omega , \tau )$ from the (polarized) Heisenberg group $\textbf{H}$ (see [35, Chap. 1] or [42, Chap. 9]), we denote the unitary operator arising from its Schrödinger representation by $\pi (x,\omega ;\tau )$. We will only be interested in $\pi (x,\omega ;0)$, which we will simply denote by $\pi (x,\omega )$.

We briefly sketch the set-up. The unitary operators we consider are time-frequency shifts which act on functions by the rule

$$\begin{aligned} \pi (x,\omega ) f(t) = M_\omega T_x f(t) = e^{2 \pi i \omega t} f(t-x). \end{aligned}$$

These give rise to Gabor systems, which we present in more detail in § 3. A Gaussian Gabor system over a lattice $\Lambda \subset \mathbb {R}^2$ is a structured function system of the form

$$\begin{aligned} \mathcal {G}(\varphi ,\Lambda ) = \{ \pi (\lambda ) \varphi \mid \lambda \in \Lambda \}, \quad \lambda = (x,\omega ) \in \mathbb {R}^2. \end{aligned}$$

The associated self-adjoint Gabor frame operator acts on functions $f \in L^2(\mathbb {R} )$ by the rule

$$\begin{aligned} S_\Lambda f = \text {vol}(\Lambda ) \sum _{\lambda \in \Lambda } \langle f, \pi (\lambda ) \varphi \rangle \, \pi (\lambda ) \varphi . \end{aligned}$$

Our main results, Theorem 1.2 and Theorem 1.3, concern the sharp spectral bounds of $S_\Lambda$. The spectral bounds of the operator will be denoted by $A_\Lambda$ and $B_\Lambda$ (we will later also pass the density of the lattice $\Lambda$ as an argument to the bounds), hence,

$$\begin{aligned} A_\Lambda \left\Vert f \right\Vert _2^2 \le \langle S_\Lambda f, f \rangle \le B_\Lambda \left\Vert f \right\Vert _2^2, \quad \forall f \in L^2(\mathbb {R} ). \end{aligned}$$

We denote the scaled von Neumann lattice and hexagonal lattice of density $\alpha > 0$ by

$$\begin{aligned} \Lambda _{1\times 1}(\alpha ) = \alpha ^{-1/2} \mathbb {Z}\times \alpha ^{-1/2} \mathbb {Z}\quad \text {and} \quad \Lambda _2(\alpha ) = \alpha ^{-1/2} \sqrt{\frac{2}{\sqrt{3}}} \begin{pmatrix} 1 &  \frac{1}{2} \\ 0 &  \frac{\sqrt{3}}{2} \end{pmatrix} \mathbb {Z}^2, \end{aligned}$$

respectively. We primarily study the behavior of the spectral or frame bound sequences $(A_\Lambda )_n$ and $(B_\Lambda )_n$ of the two Gaussian Gabor systems

$$\begin{aligned} \mathcal {G}(\varphi ,\Lambda _{1\times 1}(2^n)) \quad \text { and } \quad \mathcal {G}(\varphi , \Lambda _2(2 \cdot 3^{n-1})), \qquad n \in \mathbb {N}, \end{aligned}$$

and prove that these allow for well-established AGMs. While it has long been known that AGMs play an important role, e.g., in the theory of elliptic integrals and hypergeometric functions, their appearance in Gabor analysis is completely new. This provides a fresh connection between time-frequency analysis and the seemingly remote field of analytic number theory.

### Main results

Our main results, which are Theorem 1.2 and Theorem 1.3 below, rely on the following formulas first established by Janssen [48] for Gabor systems over rectangular lattices and their extension provided by Faulhuber [28].

#### Proposition 1.1

Let $\Lambda _{1\times 1}(2^{n}) = 2^{-n/2} \mathbb {Z}\times 2^{-n/2} \mathbb {Z}$, $n \in \mathbb {N}$, denote the scaled von Neumann lattice of density $2^n$. Denote the Gabor frame bounds of the Gabor systems $\mathcal {G}(\varphi ,\Lambda _{1\times 1}(2^n))$ by $A_{1\times 1}(2^n)$ and $B_{1\times 1}(2^n)$. Then they can be expressed by (see [48])

1.1 $$\begin{aligned} A_{1\times 1}(2\cdot 2^{n-1}) = \theta _4\left( q^{2^{n-1}}\right) ^2 \quad \text { and } \quad B_{1\times 1}(2\cdot 2^{n-1}) = \theta _3\left( q^{2^{n-1}}\right) ^2, \qquad q = e^{-\pi }. \end{aligned}$$

Let $\Lambda _2(2\cdot 3^{n-1}) = 2^{-1/2}3^{-(n-1)/2} \Lambda _2$, $n \in \mathbb {N}$, denote the scaled hexagonal lattice of density $2\cdot 3^{n-1}$. Denote the frame bounds of $\mathcal {G}(\varphi ,\Lambda _2(2\cdot 3^{n-1}))$ by $A_2(2\cdot 3^{n-1})$ and $B_2(2\cdot 3^{n-1})$. Then, they can be expressed by (see [28])

$$\begin{aligned} A_2 (2 \cdot 3^{n-1}) = b\left( q^{3^{n-1}}\right) \quad \text { and } \quad B_2(2 \cdot 3^{n-1}) = a\left( q^{3^{n-1}}\right) , \qquad q = e^{-\frac{2 \pi }{\sqrt{3}}}. \end{aligned}$$

Above, the notions $\theta _3(q)$ and $\theta _4(q)$ stand for the Jacobi theta constants (see Sect. 4.1) and *a*(*q*) and *b*(*q*) are the cubic analogs (see Sect. 4.2) in the notation of Borwein and Borwein [17].

#### Theorem 1.2

(AGM2) Let $\Lambda _{1\times 1}(2^{n}) = 2^{-n/2} \mathbb {Z}\times 2^{-n/2} \mathbb {Z}$, $n \in \mathbb {N}$, denote the scaled von Neumann lattice of density $2^n$. Denote the spectral bounds of the frame operator by $A_{1\times 1}(2^n)$ and $B_{1\times 1}(2^n)$. Then, they obey the arithmetic–geometric mean iteration:

$$\begin{aligned} B_{1\times 1}(2^{n+1}) = \frac{A_{1\times 1}(2^n)+B_{1\times 1}(2^n)}{2} \quad \text { and } \quad A_{1\times 1}(2^{n+1}) = \sqrt{A_{1\times 1}(2^n) B_{1\times 1}(2^n)}. \end{aligned}$$

The constants $A_{1\times 1}(2)$ and $B_{1\times 1}(2)$ satisfy the relation $B_{1\times 1}(2)/A_{1\times 1}(2) = \sqrt{2}$. Furthermore, denoting the classical arithmetic–geometric mean by $\textsf{ag}_2$, we have

$$\begin{aligned} A_{1\times 1}(2) = G = \frac{\Gamma \left( \frac{5}{4} \right) }{\Gamma \left( \frac{3}{2} \right) \Gamma \left( \frac{3}{4} \right) } = \frac{1}{\textsf{ag}_2\left( \sqrt{2}, 1\right) } = \theta _4(e^{-\pi })^2 = 0.834627 \ldots , \end{aligned}$$

where *G* denotes Gauss’ constant and $\theta _4$ a Jacobi theta constant.

Given the next result, note that *G* relates to the second lemniscate constant $\ell _2$ by,

$$\begin{aligned} G = \frac{1}{2 \ell _2} \quad \text { and } \quad \ell _2 = \frac{\Gamma \left( \frac{1}{2} \right) \Gamma \left( \frac{3}{4} \right) }{\Gamma \left( \frac{1}{4} \right) } = 0.599070\ldots \end{aligned}$$

More details are given in [34, Chap. 6.1].

#### Theorem 1.3

(AGM3) Let $\Lambda _2(2\cdot 3^{n-1}) = 2^{-1/2}3^{-(n-1)/2} \Lambda _2$, $n \in \mathbb {N}$, denote the scaled hexagonal lattice of density $2\cdot 3^{n-1}$ and the respective spectral bounds of the frame operator by $A_2(2\cdot 3^{n-1})$ and $B_2(2\cdot 3^{n-1})$. Then, they obey the cubic arithmetic–geometric mean iteration:

$$\begin{aligned} B_2(2\cdot 3^{n})= &   \frac{B_2(2\cdot 3^{n-1}) +2A_2(2\cdot 3^{n-1})}{3}\\  &   \quad \quad \quad \quad \quad \text {and} \\ A_2(2\cdot 3^{n})= &   \root 3 \of {A_2(2\cdot 3^{n-1}) \, \frac{A_2(2\cdot 3^{n-1}) ^2+A_2(2\cdot 3^{n-1}) B_2(2\cdot 3^{n-1}) +B_2(2\cdot 3^{n-1}) ^2}{3}} \,. \end{aligned}$$

The constants $A_2(2)$ and $B_2(2)$ satisfy the relation $B_2(2)/A_2(2) = \root 3 \of {2}$. Furthermore, denoting the cubic arithmetic–geometric mean by $\textsf{ag}_3$, we have

$$\begin{aligned} A_2(2) = \frac{1}{2 \mathcal {L}_+} = \frac{\Gamma \left( \frac{1}{6} \right) }{2 \ \Gamma \left( \frac{1}{3} \right) \Gamma \left( \frac{5}{6} \right) } = \frac{1}{\textsf {ag}_3\left( \root 3 \of {2}, 1\right) } = b\left( e^{-\frac{2 \pi }{\sqrt{3}}}\right) = 0.920371 \ldots , \end{aligned}$$

where $\mathcal {L}_+$ is the conjectural value of Landau’s constant and *b* the cubic pendant to $\theta _4^2.$

The constant $A_2(2)$ may be referred to as an equianharmonic constant [1, Chap. 18], [29]. We refer to [34, Chap. 7] and § 4.7 for more details on Landau’s constant $\mathcal {L}$. For the sake of completeness, we note that its conjectural value, obtained in [60], is

$$\begin{aligned} \mathcal {L}_+ = \frac{\Gamma \left( \frac{1}{3} \right) \Gamma \left( \frac{5}{6} \right) }{\Gamma \left( \frac{1}{6} \right) } = 0.543259 \ldots \ . \end{aligned}$$

### Remark

The connection of the spectral bounds of the Gaussian frame operator to Gauss’ constant and the conjectural value of Landau’s constant is somewhat mysterious and simply pops out of the computations. Also, to the best of our knowledge, the equality

1.2 $$\begin{aligned} \textsf{ag}_3(\root 3 \of {2}, 1) = 2 \mathcal {L}_+, \end{aligned}$$

seemed to be unknown so far and we will provide a short proof for this equation as well.

## Root systems and lattices

**Fig. 1:**
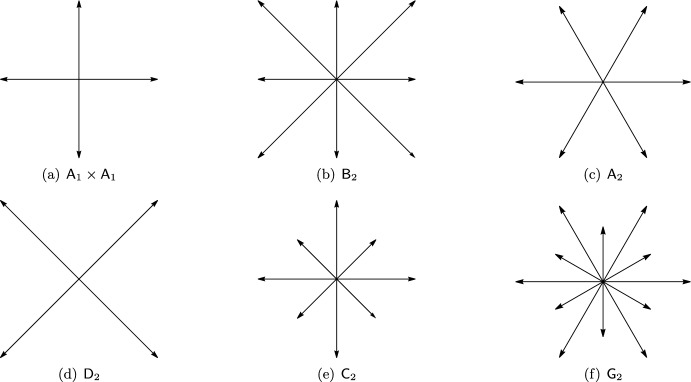
The root systems $\textsf{A}_1 \times \textsf{A}_1$ and $\textsf{D}_2$ are isomorphic as well as the root systems $\textsf{B}_2$ and $\textsf{C}_2$. All of them generate a (scaled) von Neumann lattice, by considering all integer linear combinations. The other existing root systems are $\textsf{A}_2$ and $\textsf{G}_2$. Both generate a hexagonal lattice. Note that $a A_1 \times b A_1$, $a, b > 0$ is also a root system which gives a rectangular lattice

A root system is a finite set of vectors in Euclidean space $\mathbb {R}^d$ with exceptionally high symmetries. As we are only concerned with $\mathbb {R}^2$ in this work, we start with a full list of all possible root systems in $\mathbb {R}^2$ together with their illustrations in Fig. 1.

By $v_1 \cdot v_2$ we denote the Euclidean inner product of the two vectors. A finite set *R* of vectors is called a root system if

(i) $$0 \notin R$$ and $$\text {span}(R) = \mathbb {R}^d$$.
(ii) If $v_1 \in R$, called a root, then $-v_1 \in R$ and if for $r \in \mathbb {R}$ we have $r \, v_1 \in R$, then $r=\pm 1$.
(iii) If $v_1, v_2 \in R$, then $v_2 - 2 \dfrac{v_1 \cdot v_2}{v_1 \cdot v_1} \, v_1 \in R.$
(iv) If $v_1, v_2 \in R$, then $2 \dfrac{v_1 \cdot v_2}{v_1 \cdot v_1} \in \mathbb {Z}.$

Note that in dimension 1, there is only one root system, which is called $\textsf{A}_1$ and only consists of $\{\pm 1\}$ and is contained in the only 1-dimensional lattice, which is $\mathbb {Z}$ (all up to scaling).

**Fig. 2:**
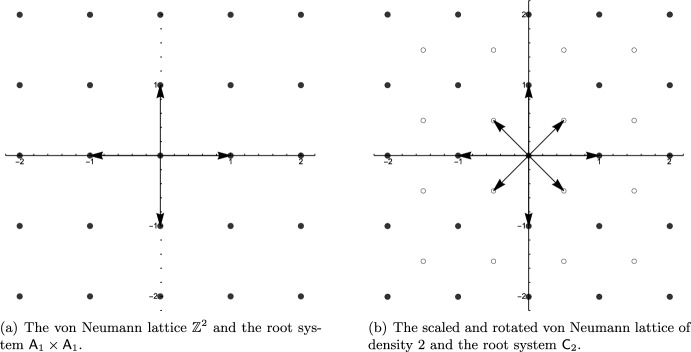
The von Neumann lattice $\mathbb {Z}^2$ contains the root system $\textsf{A}_1 \times \textsf{A}_1$. By adding new points in the center of the fundamental cell (deep hole) we obtain a lattice with twice the density. The original lattice is contained as a sub-lattice and the new lattice contains the root system $\textsf{C}_2$. The new lattice is merely a rotation of the scaled original by 45 degrees

**Fig. 3:**
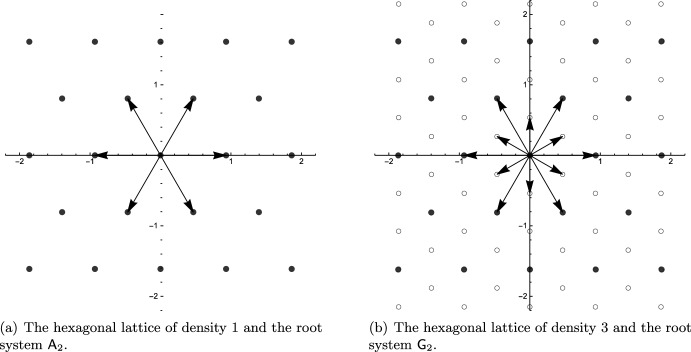
The hexagonal lattice of density 1 contains the root system $\textsf{A}_2$. By adding new points in the center of the fundamental triangle (deep hole) we obtain a lattice with thrice the density. The original lattice is contained as a sub-lattice and the new lattice contains the root system $\textsf{G}_2$. The new lattice is merely a rotation of the scaled original by 30 degrees

The 6 root systems in dimension 2 can be used to construct lattices, or, phrased differently, are contained in certain lattices. The only lattices containing root systems are the (scaled) von Neumann lattice (Fig. 2), rectangular lattices and the hexagonal lattice (Fig. 3).

In this work, we are only interested in lattices which contain a root system, which reduces our interest mainly to the von Neumann lattice and the hexagonal lattice, and to some extent to rectangular lattices. We will use the following notation for these scaled lattices, which is closely related to the $\textsf{A}_*$ notation for root systems:

- von Neumann lattice: $\Lambda _{1\times 1}(\alpha ) = \alpha ^{-1/2} \mathbb {Z}^2$,
- hexagonal lattice: $$\Lambda _2(\alpha ) = \alpha ^{-1/2} \sqrt{\dfrac{2}{\sqrt{3}}} \begin{pmatrix} 1 &  \frac{1}{2} \\ 0 &  \frac{\sqrt{3}}{2} \end{pmatrix} \mathbb {Z}^2$$,
- rectangular lattice: $\Lambda _{a \times b}(\alpha ) = \alpha ^{-1/2} \left( a \mathbb {Z}\times b \mathbb {Z}\right)$, with $a b = 1$.

In the above notation, we made the density $\alpha$ of the lattice explicit. The density is the average number of lattice points per unit area (more details are given in Sect. 3 below). Sometimes, we will also suppress the notation and simply write $\Lambda _{1\times 1}$, $\Lambda _2$ and $\Lambda _{a \times b}$, for the scaled von Neumann, hexagonal and rectangular lattices, respectively. The density, however, plays an important role in our main results, which is why we then prefer the above notation.

## Gabor systems over lattices

We consider the Hilbert space of square-integrable functions on the line, denoted by $L^2(\mathbb {R} )$. The inner product and the norm are given by

$$\begin{aligned} \langle f, g \rangle = \int _\mathbb {R}f(t) \overline{g(t)} \, dt \quad \text { and } \quad \left\Vert f \right\Vert _2^2 = \langle f, f \rangle , \quad \text { respectively}. \end{aligned}$$

The unitary operators of translation (time-shift operator) and modulation (frequency-shift operator) are given by

3.1 $$\begin{aligned} T_x f(t) = f(t-x) \quad \text { and } \quad M_\omega f(t) = e^{2 \pi i \omega t}, \quad \text { satisfying } \quad M_\omega T_x = e^{2 \pi i \omega x} T_x M_\omega , \end{aligned}$$

respectively. We recall that $\varphi (t) = 2^{1/4} e^{-\pi t^2}$ is the normalized Gaussian function. The Fourier transform of a (suitably nice) function on the line is given by

$$\begin{aligned} \mathcal {F}f(\omega ) = \int _\mathbb {R}f(t) e^{-2 \pi i \omega t} \, dt. \end{aligned}$$

Note that the Gaussian is invariant under the Fourier transform, i.e., it is an eigenfunction with eigenvalue 1: $\mathcal {F}\varphi = \varphi$. This is also true for the multi-variate Gaussians $\varphi \otimes \varphi$ and the Fourier transform over $\mathbb {R}^2$. We mention this as we will frequently use the Poisson summation formula (introduced in Sect. 4.4) with (versions of) a Gaussian of the form $\varphi \otimes \varphi$.

Combining the action of translation and modulation gives a so-called time-frequency shift, which also appears as the Schrödinger representation of an element of the (polarized) Heisenberg group ( [35, Chap. 1], [42, Chap. 9]);

$$\begin{aligned} \pi (z) = \pi (x,\omega ), \quad z=(x,\omega ) \in \mathbb {R}^2. \end{aligned}$$

In this context, we refer to $\mathbb {R}^2$ as the time-frequency plane. As the (polarized) Heisenberg group $\textbf{H}$ is a non-Abelian group, it follows that time-frequency shifts do not commute in general. This can also be seen from the fact that already $T_x$ and $M_\omega$ do not commute in general. For a nice treatise on the role of the Heisenberg group in harmonic analysis, we refer to [46]. We will let time-frequency shifts act on the Gaussian function $\varphi$:

$$\begin{aligned} \pi (z) \varphi (t) = M_\omega T_x \varphi (t) = \varphi (t-x) e^{2 \pi i \omega t} = 2^{1/4} e^{-\pi (x-t)^2} e^{2 \pi i \omega t}, \quad t \in \mathbb {R}. \end{aligned}$$

A question, going back to von Neumann in quantum mechanics [58], and studied later independently by Gabor for the purpose of communication theory [37], is whether the set

$$\begin{aligned} \mathcal {G}_0(\varphi , \mathbb {Z}^2) = \{ \pi (k,l) \varphi \mid (k,l) \in \mathbb {Z}^2\} \end{aligned}$$

is complete in $L^2(\mathbb {R} )$. Therefore, the lattice $\mathbb {Z}^2$ is often referred to as the von Neumann lattice in the context of quantum mechanics. As we now know, this is indeed the case [6, 59], (see also [43]). We also refer to [14] for a treatise on the (over)completeness of coherent states over the von Neumann lattice and its connections to zeros of theta functions.

In time-frequency analysis, one is interested in stable expansions of the form

3.2 $$\begin{aligned} f = \sum _{(k,l) \in \mathbb {Z}^2} c_{k,l} \, \pi (k,l) \varphi , \qquad \text { where } (c_{k,l}) \in \ell ^2(\mathbb {Z}^2). \end{aligned}$$

By stability, we mean that *f* is completely determined by the coefficients $(c_{k,l}) = (c_{k,l}(f))$, and the reconstruction formula (3.2) is well-behaved. In functional analytic terms, we are interested in which cases we can find a bounded coefficient operator

$$\begin{aligned} C:L^2(\mathbb {R} )\rightarrow \ell ^2(\mathbb {Z}^2),\qquad f\mapsto (c_{k,l}(f))_{(k,l) \in \mathbb {Z}^2} \end{aligned}$$

which has a bounded left inverse. For the above system $\mathcal {G}_0(\varphi , \mathbb {Z}^2)$ this is not possible, due to a manifestation of the uncertainty principle: the Balian–Low theorem [5, 53]. We will elaborate on this fact a bit later, at the end of this section.

In the above expansion (3.2), the Gaussian function $\varphi$ may be replaced by any other (suitably nice) function $g \in L^2(\mathbb {R} )$ and the index set $\mathbb {Z}^2$ by a lattice $\Lambda \subset \mathbb {R}^2$. A lattice in the time-frequency plane is a discrete co-compact subgroup of $\mathbb {R}^2$, i.e., the integer span of a basis for $\mathbb {R}^2$. We can write any lattice as

$$\begin{aligned} \Lambda = M \mathbb {Z}^2 = (v_1,v_2)\mathbb {Z}^2 = \{k v_1 + l v_2 \mid (k,l) \in \mathbb {Z}^2 \}. \end{aligned}$$

The matrix *M* contains the column vectors $v_1$ and $v_2$ which constitute a basis for $\mathbb {R}^2$, in other words, $M \in \textrm{GL}(2,\mathbb {R})$. We write $\text {vol}(\Lambda )$ for the co-volume of the lattice $\Lambda$, which is given by

$$\begin{aligned} \text {vol}(\Lambda ) = |\det (M)|, \quad \text { for } \quad \Lambda = M \mathbb {Z}^2. \end{aligned}$$

This leads to the study of Gabor systems, also known as Weyl-Heisenberg systems in mathematical physics [39], of the form:

$$\begin{aligned} \mathcal {G}(g, \Lambda ) = \{ \pi (\lambda ) g \mid \lambda \in \Lambda \}. \end{aligned}$$

The question of stable expansions of a function with respect to the Gabor system is equivalent to determining whether $\mathcal {G}(g, \Lambda )$ is a Gabor frame for $L^2(\mathbb {R} )$. The system $\mathcal {G}(g,\Lambda )$ is a frame if and only if there exist positive constants $0<A \le B < \infty$ (depending on *g* and $\Lambda$) such that

3.3 $$\begin{aligned} A \left\Vert f \right\Vert _2^2 \le \text {vol}(\Lambda ) \, \sum _{\lambda \in \Lambda } |\langle f, \pi (\lambda ) g \rangle |^2 \le B \left\Vert f \right\Vert _2^2, \quad \forall f \in L^2(\mathbb {R} ). \end{aligned}$$

This is just a spectral inequality for the self-adjoint Gabor frame operator, denoted by $S_{g,\Lambda }$:

$$\begin{aligned} S_{g,\Lambda } f  &   = \text {vol}(\Lambda ) \, \sum _{\lambda \in \Lambda } \langle f, \pi (\lambda ) g \rangle \, \pi (\lambda ) g, \quad \text { so, } \quad A \left\Vert f \right\Vert _2^2 \le \langle S_{g,\Lambda } f, f \rangle \le B \left\Vert f \right\Vert _2^2, \\    &   \quad \forall f \in L^2(\mathbb {R} ). \end{aligned}$$

Note that we have slightly adjusted the standard formulation of the frame operator and the frame inequality to an equivalent statement by multiplying by the co-volume of the lattice. This will turn out to be a convenient normalization for our main results. Also, note that the sharpest possible constants in (3.3) are actually the spectral bounds of the operator $S_{g,\Lambda }$:

$$\begin{aligned} A = \left\Vert S_{g,\Lambda }^{-1} \right\Vert _{L^2(\mathbb {R} ) \rightarrow L^2(\mathbb {R} )}^{-1}, \quad B = \left\Vert S_{g, \Lambda } \right\Vert _{L^2(\mathbb {R} ) \rightarrow L^2(\mathbb {R} )}. \end{aligned}$$

The invertibility of $S_{g,\Lambda }$ also provides a (canonical) choice of a bounded coefficient operator *C* with a bounded left inverse *D*, namely,

$$\begin{aligned} \begin{aligned} C&:L^2(\mathbb {R} )\rightarrow \ell ^2(\Lambda ),\qquad f\mapsto (\langle f, \pi (\lambda )S^{-1}_{g,\Lambda } g\rangle )_{\lambda \in \Lambda } \\ D&:\ell ^2(\Lambda )\rightarrow L^2(\mathbb {R} ),\qquad (c_{\lambda })_{\lambda \in \Lambda } \mapsto \sum \limits _{\lambda \in \Lambda } c_\lambda \pi (\lambda ) g. \end{aligned} \end{aligned}$$

We will now briefly come back to the above-mentioned manifestation of the uncertainty principle in time-frequency analysis. The quantity $\text {vol}(\Lambda )^{-1}$ is called the density of the lattice and gives the average number of points (or available information) per unit area. For Gaussian Gabor systems of the form $\mathcal {G}(\varphi , \Lambda )$, we simply write $S_\Lambda$ for the frame operator. In this case, we know that the necessary density condition $\text {vol}(\Lambda ) < 1$, imposed by the Balian–Low theorem, is already sufficient for the Gabor system to produce a Gabor frame:

$$\begin{aligned} \mathcal {G}(\varphi , \Lambda ) \text { is a frame} \quad \Longleftrightarrow \quad \text {vol}(\Lambda ) < 1. \end{aligned}$$

This is a consequence of the celebrated results of Lyubarskii [54], Seip [62], Seip and Wallstén [63]. The condition $\text {vol}(\Lambda ) < 1$ tells us that we need (a bit) more than 1 time-frequency sample per unit area. This is comparable to the Nyquist rate in the classical Whittaker-Koltelnikov-Shannon sampling theorem for band-limited functions [51, 64, 69].

## AGMs and lattice theta functions

The arithmetic–geometric means (AGMs) of order *N* are recursive constructions of sequences studied by Borwein and Borwein [17]. The general iteration for $1 < N\in \mathbb {N}$ is initialized with two starting values $a_0$ and $b_0$, which are allowed to be complex numbers. We will only use the process for real values $a_0> b_0 > 0$. Three sequences are defined recursively:

$$\begin{aligned} a_{n+1}=\frac{a_n+(N-1)b_n}{N}, \qquad c_{n+1}=\frac{a_n-b_n}{N}, \qquad \text {where}\quad b_n^N=a_n^N-c_n^N. \end{aligned}$$

Then the sequences $\left( a_n\right) _{n\in \mathbb {N}}$ and $\left( b_n\right) _{n\in \mathbb {N}}$ converge to a common limit, denoted by $\textsf{ag}_N(a,b)$, with convergence rate *N*, best justified by

$$\begin{aligned} a_{n+1}^N-b_{n+1}^N = \left( \frac{a_n-b_n}{N}\right) ^N. \end{aligned}$$

We will consider the arithmetic–geometric means of order 2 and 3, with particular sequences. By substituting $c_n$, we can describe $(a_n)_{n\in \mathbb {N}}$ and $(b_n)_{n\in \mathbb {N}}$ independently of $(c_n)_{n\in \mathbb {N}}$ as:

$$\begin{aligned} a_{n+1}&= \frac{a_n+b_n}{2}&\qquad \text {and}\qquad b_{n+1}&=\sqrt{a_n b_n},&\qquad \qquad (N=2), \\ a_{n+1}&= \frac{a_n+2b_n}{3}&\qquad \text {and}\qquad b_{n+1}&=\root 3 \of {b_n\left( \frac{a_n^2+a_nb_n+b_n^2}{3}\right) },&\qquad \qquad (N=3). \end{aligned}$$

The case $N = 2$ simply leads to the classical arithmetic–geometric mean. This has already been studied intensively by Gauss, who proved that it can be used to exactly and efficiently compute elliptic integrals [50, entry 98, entry 102] (see also [23] and [38, pp. 446 ff.]). Note that, for $x > 0$, it is obvious from the above iterations for $N=2$ and $N=3$ that $\textsf{ag}_2$ and $\textsf{ag}_3$ are homogeneous: $\quad \textsf{ag}_2(x a_0, x b_0) = x \, \textsf{ag}_2(a_0, b_0) \quad \text { and } \quad \textsf{ag}_3(x a_0, x b_0) = x \, \textsf{ag}_3(a_0, b_0).$

### Jacobi theta functions

Theta functions are classical objects, appearing in many branches of mathematics and also in other sciences. We refer to the textbooks [65, 70] for proper introductions as well as to [21, Chap. 4] for their connection with sphere packings and coverings and related topics. Still, we want to clarify the notation which we use in this work. We write the Jacobi theta functions in the following way;

$$\begin{aligned} \vartheta _2(z;q) = \sum _{k \in \mathbb {Z}} q^{(k+\frac{1}{2})^2} e^{2 \pi i (k+ \frac{1}{2}) z}, \quad \vartheta _3(z;q) = \sum _{k \in \mathbb {Z}} q^{k^2} e^{2 \pi i k z}, \\\quad \vartheta _4(z;q) = \sum _{k \in \mathbb {Z}} (-1)^k q^{k^2} e^{2 \pi i k z}, \end{aligned}$$

where $z \in \mathbb {C}$ and $0< \left| q\right| < 1$. It is also common to replace the nome *q* by $e^{\pi i \tau }$. Then $\tau$ needs to be chosen from the Siegel upper half space

$$\begin{aligned} \mathbb {H} = \{ z \in \mathbb {C}\mid \Im (z) > 0 \}, \end{aligned}$$

in order to ensure convergence of the series. The $\vartheta$-functions are entire for $z \in \mathbb {C}$ and holomorphic for $\tau \in \mathbb {H}$. For our purposes, it suffices to consider the functions for $z = 0$, which carry the name theta constants. We write

$$\begin{aligned} \theta _m(q) = \vartheta _m(0,q), \quad m \in \{2,3,4\}. \end{aligned}$$

We remark that there is also the (prototype) Jacobi theta function

$$\begin{aligned} \vartheta _1(z,q) = -i \sum _{k \in \mathbb {Z}} (-1)^k q^{(k+\frac{1}{2})^2} e^{2 \pi i (k + \frac{1}{2}) z}. \end{aligned}$$

It is an odd function in *z*: its theta constants vanish identically and are not of interest here.

### The analogs

In their paper on the cubic AGM [17], Borwein and Borwein introduced new $\theta$-like functions. Following the notation in [17], we set

$$\begin{aligned} a(q)= &   \sum _{m,n \in \mathbb {Z}} q^{m^2+mn+n^2},\\ b(q)= &   \sum _{m,n \in \mathbb {Z}} \zeta ^{n-m} q^{m^2+mn+n^2} \quad \text { and } \quad c(q) = \sum _{m,n \in \mathbb {Z}} q^{(m+\frac{1}{3})^2+(m+\frac{1}{3})(n+\frac{1}{3})+(n+\frac{1}{3})^2}, \end{aligned}$$

where $\zeta ^3=1$, $\zeta \ne 1$ and $0< \left| q\right| < 1$. The analogy is understood through the correspondence

$$\begin{aligned} \begin{aligned} \theta _3(q)^2 \ \longleftrightarrow \ a(q), \qquad \theta _4(q)^2 \ \longleftrightarrow \ b(q), \qquad \theta _2(q)^2 \ \longleftrightarrow \ c(q). \end{aligned} \end{aligned}$$

Note that the cubic analogs can also be expressed by Lambert series (so single series instead of double series), see e.g., [17] or [55]. This facilitates numerical computations.

### The AGM of theta functions

The triples of theta constants and their cubic analogs satisfy (see [21, Chap. 4.4] for the classical theta functions and [17] for the cubic analogs)

4.1 $$\begin{aligned} \begin{aligned} 2\theta _3(q^2)^2&= \theta _3(q)^2+\theta _4(q)^2&\longleftrightarrow&\qquad&3a(q^3)&= a(q) + 2b(q),  &   \\ 2\theta _2(q^2)^2&=\theta _3(q)^2 - \theta _4(q)^2 \qquad&\longleftrightarrow&\qquad&3c(q^3)&= a(q) - b(q),  &   \\ \theta _3(q)^4&= \theta _4(q)^4+\theta _2(q)^4 \qquad&\longleftrightarrow&\qquad&a(q)^3&= b(q)^3 + c(q)^3.  &   \\ \end{aligned} \end{aligned}$$

This shows that $\theta _3^2,\,\theta _4^2,\,\theta _2^2$ and $a,\,b,\,c$ fit within the $\textsf{ag}_2$ and $\textsf{ag}_3$ construction, respectively. For more identities involving the cubic analogs we refer to the articles [18] and [45].

### Lattice theta functions and Gaussian lattice sums

Recall that in order for the Gabor system $\mathcal {G}(\varphi , \Lambda )$ to be a frame, the frame inequality has to be satisfied:

$$\begin{aligned} A_\Lambda \left\Vert f \right\Vert _2^2 \le \text {vol}(\Lambda ) \, \sum _{\lambda \in \Lambda } |\langle f, \pi (\lambda ) \varphi \rangle |^2 \le B_\Lambda \left\Vert f \right\Vert _2^2, \quad \forall f \in L^2(\mathbb {R} ). \end{aligned}$$

Finding extremal functions *f* (depending on $\Lambda$) such that the sharp bounds are met is a cumbersome task. We may relax the problem by only considering the set $\{\pi (z) \varphi \mid z \in \mathbb {R}^2 \}$, which is known to be dense in $L^2(\mathbb {R} )$ [42, Chap. 1.5]. Noting that $\left\Vert \pi (z) \varphi \right\Vert _2^2 = 1$, we have:

4.2 $$\begin{aligned} A_\Lambda \le \text {vol}(\Lambda ) \, \sum _{\lambda \in \Lambda } |\langle \pi (z) \varphi , \pi (\lambda ) \varphi \rangle |^2 \le B_\Lambda . \end{aligned}$$

A small computation shows that we have (see also [35, Prop. (1.48)], [42, Chap. 1.5])

$$\begin{aligned} |\left\langle \pi (z) \varphi , \pi (\lambda ) \varphi \right\rangle |^2 = |\langle \varphi , \pi (\lambda -z) \varphi \rangle |^2 = e^{{-\pi }|\lambda -z|^2}. \end{aligned}$$

Since $\Lambda$ has an additive group structure, we see that $\lambda \in \Lambda$ if and only if $-\lambda \in \Lambda$. So, we can actually re-write (4.2) as

$$\begin{aligned} A_\Lambda \le \text {vol}(\Lambda ) \sum _{\lambda \in \Lambda } e^{-\pi |\lambda +z|^2} \le B_\Lambda , \quad \forall z \in \mathbb {R}^2. \end{aligned}$$

It should be noted that the above inequality may actually not become sharp.

For a lattice $\Lambda$ of unit density, i.e., $\text {vol}(\Lambda ) = 1$, and $\alpha >0$, we now introduce the following family of lattice theta functions:

$$\begin{aligned} \theta _{\Lambda }(b;\alpha )= \sum _{\lambda \in \Lambda } e^{-\pi \alpha |\lambda +b|^2}, \quad b \in \mathbb {R}^2. \end{aligned}$$

These lattice theta functions, which are Gaussian lattice sums shifted by $b=(b_1,b_2)$ in $\mathbb {R}^2$, will play a central role in this part. Actually, it is more their symplectic dual, the modulated Gaussian lattice sums, which will be of importance. For a lattice of unit density, this is

$$\begin{aligned} \widehat{\theta }_\Lambda (b;\alpha ) = \sum _{\lambda \in \Lambda } e^{-\pi \alpha |\lambda |^2} e^{2 \pi i \sigma (b, \lambda )}. \end{aligned}$$

At this point, it is necessary to introduce the symplectic form $\sigma (. \,, \,. )$ and the (symplectic) Poisson summation formula. For a suitable function *f* the Poisson summation formula for a lattice $\Lambda$ and its dual lattice $\Lambda ^\perp$ is (see [42, Chap. 1])

$$\begin{aligned} \sum _{\lambda \in \Lambda } f(\lambda +x) = \text {vol}(\Lambda )^{-1} \sum _{\lambda ^\perp \in \Lambda ^\perp } \mathcal {F}f(\lambda ^\perp ) e^{2 \pi i \lambda ^\perp \cdot x}. \end{aligned}$$

We only give a characterization of the dual lattice in dimension 2, but the statement is easily transferred to higher dimensions. Denoting by $M^{-T}$ the inverse of *M* transposed we have

$$\begin{aligned} \Lambda ^\perp = \{ \lambda ^\perp \in \mathbb {R}^2 \mid \lambda ^\perp \cdot \lambda \in \mathbb {Z}, \, \forall \lambda \in \Lambda \} = M^{-T} \mathbb {Z}^2, \quad \Lambda = M \mathbb {Z}^2. \end{aligned}$$

As we are working in dimension 2, we can actually exploit the symplectic structure of the time-frequency plane (see [35, 39]). We introduce the standard symplectic form $\sigma$, which is skew-symmetric and will replace the Euclidean inner produce in some computations;

$$\begin{aligned} \sigma (z,z') = z_1 z_2' - z_2 z_1' = z \cdot \mathcal {J} z', \quad z=(z_1,z_2), \, z'=(z_1',z_2') \in \mathbb {R}^2. \end{aligned}$$

We denote by $\mathcal {J}$ the standard symplectic matrix. In $\mathbb {R}^2$ it is simply a rotation by 90 degrees;

$$\begin{aligned} \mathcal {J} = \begin{pmatrix} 0 &  1\\ -1 &  0 \end{pmatrix}. \end{aligned}$$

As we work in $\mathbb {R}^2$, we can use $\sigma$ to define the symplectic Fourier transform [39, 40]:

$$\begin{aligned} \mathcal {F}_\sigma F(z) = \iint _{\mathbb {R}^2} F(z') e^{2 \pi i \sigma (z,z')} \, dz'. \end{aligned}$$

The symplectic Fourier transform carries many properties of the ordinary Fourier transform. It is for example unitary. The main difference is that it is involutive, i.e.,

$$\begin{aligned} \mathcal {F}_\sigma (\mathcal {F}_\sigma (F)) = F. \end{aligned}$$

Using the symplectic machinery, we can easily introduce a version of the Poisson summation formula for lattices in $\mathbb {R}^2$. We call it the symplectic Poisson summation formula:

$$\begin{aligned} \sum _{\lambda \in \Lambda } F(\lambda +z) = \text {vol}(\Lambda )^{-1} \sum _{\lambda ^\circ \in \Lambda ^\circ } \mathcal {F}_\sigma F(\lambda ^\circ ) e^{2 \pi i \sigma (\lambda ^\circ , z)}, \quad z \in \mathbb {R}^2. \end{aligned}$$

Here, $\Lambda ^\circ$ denotes the adjoint or symplectic dual lattice. This is just the usual dual lattice rotated by 90 degrees. It is characterized by commuting time-frequency shifts:

$$\begin{aligned} \Lambda ^\circ = \{\lambda ^\circ \in \mathbb {R}^2 \mid \pi (\lambda )\pi (\lambda ^\circ ) = \pi (\lambda ^\circ ) \pi (\lambda ), \; \forall \lambda \in \Lambda \} = \mathcal {J} M^{-T} \mathbb {Z}^2. \end{aligned}$$

It should be noted that for 2-dimensional lattices the adjoint is actually simply a re-scaling of the lattice $\Lambda$, i.e., for $\Lambda (\alpha ) = \alpha ^{-1/2} M \mathbb {Z}^2$, with $M \in \textrm{SL}(2,\mathbb {R})$ it holds that

$$\begin{aligned} \Lambda ^\circ = \alpha \Lambda . \end{aligned}$$

We have the following functional equation, similar to the Jacobi identity, which follows from the symplectic Poisson summation formula:

$$\begin{aligned} \theta _\Lambda (b;\alpha ) = \tfrac{1}{\alpha } \widehat{\theta }_\Lambda (b;\tfrac{1}{\alpha }). \end{aligned}$$

The families of functions $\theta _\Lambda (b;\alpha )$ and $\widehat{\theta }_\Lambda (b;\alpha )$ have been studied thoroughly by Bétermin and Faulhuber for the special argument ${\widetilde{b}}=(1/2,1/2)$, where $b = M \, {\widetilde{b}}$, $\Lambda = M \mathbb {Z}^2$, $M \in \textrm{SL}(2,\mathbb {R})$, is the center of the fundamental cell of the lattice [11]. It should be evident that we use column vectors in $\mathbb {R}^2$, even though we write them as row vectors. Bétermin, Faulhuber and Steinerberger studied the case of *b* being the minimizer of the lattice theta function [12]. The subtlety in [12] is that, in general, the minimizer depends on $\alpha$. In both cases, the hexagonal lattice turns out to be the global maximizer among lattices of unit density and all $\alpha > 0$, which gives a dual universal optimality result among lattices in the spirit of [20]. The case $b=(0,0)$, which can be replaced by any lattice point and which is the maximizer of the lattice theta functions, has been fully treated by Montgomery [56], proving that the hexagonal lattice is the unique minimizer in this case. In all cases, i.e., in [11, 12, 56], the scaled von Neumann lattice is a critical point and, indeed, it is a saddle point in the set of lattices. These are a few reasons why these lattices are of special interest. For more details on the connection to Gabor frames, we refer to [28] and [31]. For a more detailed discussion on the parametrization of lattices and symplectic methods for theta functions, we refer to [11].

### The fundamental identity of Gabor analysis

It is now advantageous to introduce the following notation, making the volume of the lattice explicit when needed:

$$\begin{aligned} \Lambda (\alpha ) = \alpha ^{1/2} M \mathbb {Z}^2, \quad M \in \textrm{SL}(2,\mathbb {R}), \; \alpha > 0. \end{aligned}$$

We have the simple consequence that $\text {vol}(\Lambda (\alpha )) = \alpha .$ Note, that we can write $\Lambda (\alpha ) = \alpha ^{1/2} \Lambda (1)$. This provides the following significant number theoretic relation between Gaussian lattice sums and Gaussian Gabor frame bounds:

4.3 $$\begin{aligned} A_\Lambda \le \text {vol}(\Lambda (\alpha ))\, \sum _{\lambda \in \Lambda (\alpha )} e^{{-\pi }|\lambda +z|^2} = \alpha \, \theta _{\Lambda (1)}(z;\alpha ^{1/2}) \le B_\Lambda , \quad z \in \mathbb {R}^2. \end{aligned}$$

Next, we introduce the fundamental identity of Gabor analysis (FIGA), which is the Poisson summation formula in disguise (suppressing the dependency of $\Lambda$ on the volume $\alpha$ again):

4.4 $$\begin{aligned} \text {vol}(\Lambda ) \sum _{\lambda \in \Lambda } \langle f_1, \pi (\lambda ) g_1 \rangle \overline{\langle f_2, \pi (\lambda ) g_2 \rangle } = \sum _{\lambda ^\circ \in \Lambda ^\circ } \langle g_2, \pi (\lambda ) g_1 \rangle \overline{\langle f_2, \pi (\lambda ) f_1 \rangle }. \end{aligned}$$

We refer to [32, 41] or [49] for details and when the formula is applicable. As we use the Gaussian window $\varphi$, all requirements are, however, met. Using (4.4), we get

$$\begin{aligned} \text {vol}(\Lambda ) \, \sum _{\lambda \in \Lambda } |\langle f, \pi (\lambda ) \varphi \rangle |^2&= \text {vol}(\Lambda ) \sum _{\lambda \in \Lambda } \langle f, \pi (\lambda ) \varphi \rangle \, \overline{\langle f, \pi (\lambda ) \varphi \rangle }\\&= \sum _{\lambda ^{\circ } \in \Lambda ^{\circ }} \langle \varphi , \pi (\lambda ^\circ ) \varphi \rangle \, \overline{\langle f, \pi (\lambda ^\circ ) f \rangle }\\&\le \sum _{\lambda ^{\circ } \in \Lambda ^{\circ }} |\langle \varphi , \pi (\lambda ^\circ ) \varphi \rangle | \, |\overline{\langle f, \pi (\lambda ^\circ ) f \rangle }|\\&\le \sum _{\lambda ^{\circ } \in \Lambda ^{\circ }} |\langle \varphi , \pi (\lambda ^\circ ) \varphi \rangle | \, \left\Vert f \right\Vert _2^2. \end{aligned}$$

In the last step, we used the fact that $|\langle f, \pi (\lambda ) f \rangle | \le |\langle f, f \rangle |=\left\Vert f \right\Vert _2^2$, which follows from the Cauchy-Schwarz inequality (see [42, Lem. 4.2.1]). It readily follows (see also [67]) that

4.5 $$\begin{aligned} B_\Lambda \left\Vert f \right\Vert _2^2 \le {\widetilde{B}}_\Lambda \left\Vert f \right\Vert _2^2, \quad \text { where } \quad {\widetilde{B}}_\Lambda = \sum _{\lambda ^{\circ } \in \Lambda ^{\circ }} |\langle \varphi , \pi (\lambda ^\circ ) \varphi \rangle | = \sum _{\lambda ^\circ \in \Lambda ^\circ } e^{-\frac{\pi }{2} |\lambda ^\circ |^2}. \end{aligned}$$

Hence, the quantity ${\widetilde{B}}_\Lambda$ is a Bessel bound (not necessarily the sharpest one) for the Gabor system $\mathcal {G}(\varphi , \Lambda )$ and we have $B_\Lambda \le {\widetilde{B}}_\Lambda$. Combining observations (4.3) and (4.5), we conclude:

$$\begin{aligned} A_\Lambda \le \text {vol}(\Lambda )\, \sum _{\lambda \in \Lambda } e^{{-\pi }|\lambda +z|^2}= \alpha \, \theta _{\Lambda (1)}(z;\alpha ^{1/2}) \le B_\Lambda \le {\widetilde{B}}_\Lambda , \quad \forall z \in \mathbb {R}^2. \end{aligned}$$

### Ramanujan’s corresponding theories

In this part, we explain a nice connection between Gaussian lattice sums, lattice theta function and Ramanujan’s corresponding theories of signature 2 and 3. This connection will play a central role in the present work and is a reason for the number theoretic character of Gaussian Gabor frames. The main references for the section are the Ramanujan notebooks edited by Berndt, in particular, [8, Chap. 17] for the von Neumann lattice (theory of signature 2) and [9, Chap. 33] for the hexagonal lattice (corresponding theory of signature 3). The part of the corresponding theory which we need has been put on solid ground by Borwein and Borwein [17] (see also [16]).

For what follows, we need to introduce Gauss’ hypergeometric function $$ _{2}{F}_{1}$$. For $$n \in \mathbb {Z}$$, let the rising Pochhammer symbol be denoted by

$$\begin{aligned} (z)_n =\frac{\Gamma (z+n)}{\Gamma (z)},\ z \in \mathbb {C}, \end{aligned}$$

where $\Gamma (z)$ is Euler’s Gamma function;

$$\begin{aligned} \Gamma (z)=\int _{\mathbb {R}_{+}}t^{z-1}e^{-t}\ dt,\,\,\, \text{ for }~ \textrm{Re}(z)>0. \end{aligned}$$

In this work, we will consider the case of Gauss’ hypergeometric function with positive parameters *a*, *b*, *c* and real variable $0<x<1$, defined by

$$\begin{aligned}  _{2}{F}_{1}(a,b; c; x)=\sum _{n=0}^{\infty } \frac{(a)_n(b)_n}{(c)_n}\frac{x^n}{n!}. \end{aligned}$$

The parameters fulfill $a+b \le c$ and $x \in (0,1)$, which means that we do not run into convergence issues. For the sake of completeness, and to justify the name *elliptic modulus*, we introduce the complete elliptic integral of the first kind, *K*, and refer to the textbook of Whittaker and Watson [70, Chap. 22.3]:

$$\begin{aligned} K(k) = \int _{0}^{\pi /2} \frac{d\varphi }{\sqrt{1-k^2\sin ^2\varphi }}. \end{aligned}$$

Here, $0<k<1$ is the elliptic modulus of *K*. The elliptic modulus is also defined by means of the Jacobian elliptic functions, i.e., the theta constants, as

$$\begin{aligned} k(q)=\frac{\theta _2(q)^2}{\theta _3(q)^2}, \quad 0< |q| < 1. \end{aligned}$$

Obviously, it depends upon $q=e^{\pi i \tau },\ \tau \in \mathbb {H}$, but we will suppress this dependence in the sequel. The complementary elliptic modulus is denoted by $k'$ (and depends on *q*). It is defined by the property

$$\begin{aligned} k^2+k'^2=1 \quad \Longleftrightarrow \quad \frac{\theta _2(q)^4}{\theta _3(q)^4} + \frac{\theta _4(q)^4}{\theta _3(q)^4} = 1. \end{aligned}$$

The next formula is quite remarkable and was already known to Gauss

4.6 $$\begin{aligned}  &   \frac{2}{\pi } \, K(k) = \frac{2}{\pi } \, \int _0^{\pi /2} \frac{d \varphi }{\sqrt{1 - k^2 \sin (\varphi )^2}} = \sum _{n=0}^\infty \frac{\left( \tfrac{1}{2} \right) _n^2}{n!} \frac{k^{2n}}{n!} \nonumber \\    &   \quad =  _2F_1( \tfrac{1}{2},\tfrac{1}{2}, 1, k^2) = \frac{1}{\textsf{ag}_2(k',1)}. \end{aligned}$$

A proof, which is essentially the original proof of Gauss with some details filled in by Jacobi, is given in [16, Chap. 1.2] and [23]. The following result is not needed immediately for our purpose, but it will be essential in proving (1.2), i.e., that the conjectural value $\mathcal {L}_+$ of Landau’s constant can be obtained as a cubic arithmetic–geometric mean (see [52] for the problem and [60] for the conjectural solution). The formula was known to Gauss and it can be found as Entry 34 in the textbook of Berndt [7, Chap. 10];

4.7 $$\begin{aligned}  _2F_1 \left( a,b; \tfrac{1}{2} (a+b+1); \tfrac{1}{2} \right) = \sqrt{\pi } \, \frac{\Gamma \left( \tfrac{a+b+1}{2} \right) }{\Gamma \left( \tfrac{a+1}{2} \right) \Gamma \left( \tfrac{b+1}{2} \right) }. \end{aligned}$$

Finally, we arrive at a kind of inversion formula, which was established by Ramanujan (see [8, Entry 3, Chap. 17]). Let $k = \theta _2^2/\theta _3^2$ be the elliptic modulus from above, then

4.8 $$\begin{aligned}  _2F_1 \left( \tfrac{1}{2},\tfrac{1}{2}; 1; k^2 \right) = \theta _3(q)^2. \end{aligned}$$

Note that this connects the theta constant to the arithmetic–geometric mean, and the complete elliptic integral of the first kind by (4.6).

The Eq. (4.8) is a statement about lattice theta functions: $\theta _2(e^{-\pi \alpha })^2 = \theta _{\Lambda _{1\times 1}}(b_\circ ; \alpha )$ and $b_\circ = (1/2, 1/2)$, yielding the deep hole in the von Neumann lattice, which is also the minimizer of the $\theta _{\Lambda _{1\times 1}}$ for any fixed $\alpha > 0$ (see [13, 31, 48]). We remark that a deep hole is a point which globally maximizes the distance to the closest lattice point (local maximizers are called shallow holes) [21, Chap. 1]. Evaluating $\theta _{\Lambda _{1\times 1}}$ at a lattice point, which always is a maximizer (see [12]), we see that $\theta _3(e^{-\pi \alpha })^2 = \theta _{\Lambda _{1\times 1}}(0;\alpha )$. So, the elliptic modulus is

$$\begin{aligned} k(e^{-\pi \alpha }) = \frac{\theta _{\Lambda _{1\times 1}}(b_\circ ; \alpha )}{\theta _{\Lambda _{1\times 1}}(0;\alpha )}, \end{aligned}$$

which is the ratio of the global minimum and maximum of the lattice theta function over a scaled von Neumann lattice. Gauss’ hypergeometric function separates the minimum and maximum from each other.

Next, we show that the theory of signature 3 is intimately related to the lattice theta function over the hexagonal lattice $\Lambda _2= H\mathbb {Z}^2$, where

$$\begin{aligned} H = \tfrac{\sqrt{2}}{\root 4 \of {3}} \begin{pmatrix} 1 & \quad \frac{1}{2} \\ 0 & \quad \frac{\sqrt{3}}{2} \end{pmatrix} \end{aligned}$$

is the generating matrix for the lattice $\Lambda _2$. It is pertinent to note that sometimes $\Lambda _2$ is called a triangular lattice since half of its fundamental domain is an equilateral triangle, while the name “hexagonal lattice" is related to the fact that its Voronoi cells are all regular hexagons. The Voronoi cell of a lattice point is the set of all points which are closer to the lattice point than to any other point in the lattice (see [21, Chap. 1]).

For $\alpha > 0$, $c = H {\widetilde{c}} = H({\widetilde{c}}_1,{\widetilde{c}}_2)$, the hexagonal lattice theta function is explicitly given by

$$\begin{aligned} \theta _{\Lambda _2}(c;\alpha ) = \sum _{\lambda \in \Lambda _2} e^{-\pi \alpha |\lambda + c|^2} = \sum _{(k,l) \in \mathbb {Z}^2} e^{\frac{-2\pi }{\sqrt{3}} ( (k+{\widetilde{c}}_1)^2 + (k+{\widetilde{c}}_1)(l+{\widetilde{c}}_2) + (l+{\widetilde{c}}_2)^2)}. \end{aligned}$$

Our next aim is to provide another significant relation between the hexagonal lattice theta function and the hypergeometric function, which will be obtained by Ramanujan’s corresponding theory of signature 3. Ramanujan’s corresponding theories of signature *r* rely on

$$\begin{aligned}  _{2}{F}_{1}\big (\tfrac{1}{r},\tfrac{r-1}{r}; 1; . \big ), \quad \text { for } r \in \{2,3,4,6\}. \end{aligned}$$

This theory of signature 3 involves the cubic analogs of the squares of Jacobi’s theta functions denoted as *a*(*q*), *b*(*q*) and *c*(*q*) as introduced in Sect. 4.2. The cubic analog of the elliptic modulus *k*(*q*) and the complementary elliptic modulus $k'(q)$ are given by

$$\begin{aligned} s(q) = \frac{c(q)}{a(q)} \quad \text { and } \quad s'(q) = \frac{b(q)}{a(q)}, \quad \text { fulfilling } \quad s(q)^3 + s'(q)^3 = 1. \end{aligned}$$

The theory was put on a solid mathematical basement in [17] and we also find the following formula [17, Thm. 2.1(b) & Thm. 2.3] (see also [9, Chap. 33], [10, Lem. 2.6], or [19]):

4.9 $$\begin{aligned}  _{2}{F}_{1}\Big (\tfrac{1}{3},\tfrac{2}{3}; 1; s^3\Big ) = \frac{1}{\textsf{ag}_3(1,s')} = a(q). \end{aligned}$$

The statement of [17, Thm. 2.1(b)] contains a harmless typo, which becomes apparent when looking at the proof. Note that (4.9) is a statement on hexagonal lattice theta functions, as we have the following relations between the cubic analogs and hexagonal lattice theta functions:

$$\begin{aligned} a(q) = \theta _{\Lambda _2}(0;\alpha ), \quad b(q) = \widehat{\theta }_{\Lambda _2}(c_\circ ;\alpha ), \quad c(q) = \theta _{\Lambda _2}(c_\circ ,\alpha ), \qquad q= e^{- \frac{2 \pi }{\sqrt{3}} \alpha }. \end{aligned}$$

The point $c_\circ = H(1/3,1/3)$ is a deep hole of the hexagonal lattice. Moreover, for all $\alpha >0$, the deep hole is the minimizer of the $\theta _{\Lambda _2}$ and $\widehat{\theta }_{\Lambda _2}$. This shows the intimate relation of the family of hexagonal lattice theta functions to Ramanujan’s corresponding theory of signature 3.

### The constants *G* and $\mathcal {L}$ and proof of Equation (1.2)

In this section, we prove (1.2) from the Remark. We start by recalling that the conjectural value of Landau’s constant $\mathcal {L}$, presented in [52], is the following value found in [60];

$$\begin{aligned} \mathcal {L}_+ = \frac{\Gamma (\frac{1}{3}) \Gamma (\frac{5}{6})}{\Gamma (\frac{1}{6})} = 0.543259 \ldots \, . \end{aligned}$$

It is known that $\frac{1}{2} < \mathcal {L} \le \mathcal {L}_+$ and it is conjectured that the second inequality is sharp.

#### Proof of (1.2)

We use Gauss’ formula (4.7), connecting the hypergeometric function $$ _2F_1$$ with the ratio of Gamma functions. The specific values we need are $$a=1/3$$ and $$b=2/3$$:

4.10 $$\begin{aligned}  _2F_1(\tfrac{1}{3},\tfrac{2}{3};1;\tfrac{1}{2}) = \sqrt{\pi } \frac{\Gamma (1)}{\Gamma (\frac{2}{3}) \Gamma (\frac{5}{6})} = \frac{\Gamma (\frac{1}{2})}{\Gamma (\frac{2}{3}) \Gamma (\frac{5}{6})}. \end{aligned}$$

Now, we use Legendre’s duplication formula for the Gamma function [1, 6.1.18]:

$$\begin{aligned} \Gamma (2z) = 2^{2z-1} \pi ^{-1/2} \, \Gamma (z) \Gamma (z+\tfrac{1}{2}). \end{aligned}$$

Evaluating at $z= 1/6$ and some simple manipulations yield

$$\begin{aligned} \Gamma (\tfrac{2}{3}) = 2^{2/3} \frac{\Gamma (\frac{1}{2}) \Gamma (\frac{1}{3})}{\Gamma (\frac{1}{6})}. \end{aligned}$$

Substituting $\Gamma (2/3)$ in (4.10) by the above expression then gives

$$\begin{aligned}  _2F_1(\tfrac{1}{3},\tfrac{2}{3};1;\tfrac{1}{2}) = 2^{-2/3} \frac{\Gamma (\frac{1}{6})}{\Gamma (\frac{1}{3}) \Gamma (\frac{5}{6})} = \frac{1}{2^{2/3} \mathcal {L}_+}. \end{aligned}$$

By using the (symplectic) Poisson summation formula, we see that $b(e^{-\frac{2 \pi }{\sqrt{3}}}) = c(e^{-\frac{2 \pi }{\sqrt{3}}})$ and by using the cubic identity, we find

$$\begin{aligned} a(e^{-\frac{2 \pi }{\sqrt{3}}})^3 = b(e^{-\frac{2 \pi }{\sqrt{3}}})^3 + c(e^{-\frac{2 \pi }{\sqrt{3}}})^3 = 2 b(e^{-\frac{2 \pi }{\sqrt{3}}})^3 \quad \Longrightarrow \quad a(e^{-\frac{2 \pi }{\sqrt{3}}}) = 2^{1/3} b(e^{-\frac{2 \pi }{\sqrt{3}}}). \end{aligned}$$

Moreover, we have

$$\begin{aligned} s(e^{-\frac{2 \pi }{\sqrt{3}}})^3 = s'(e^{-\frac{2 \pi }{\sqrt{3}}})^3 = \frac{1}{2}. \end{aligned}$$

Plugging this into (4.9) we obtain

$$\begin{aligned}  _2F_1(\tfrac{1}{3},\tfrac{2}{3};1;\tfrac{1}{2}) = a(e^{-\frac{2 \pi }{\sqrt{3}}}) = 2^{-2/3} \frac{\Gamma (\frac{1}{6})}{\Gamma (\frac{1}{3}) \Gamma (\frac{5}{6})} = \frac{1}{2^{2/3} \mathcal {L}_+} = \frac{1}{\textsf{ag}_3(1,2^{-1/3})}. \end{aligned}$$

So, we already arrived at the equality $2^{2/3} \mathcal {L}_+ = \textsf{ag}_3\left( 1, 2^{-1/3}\right)$. Now, we use that $\textsf{ag}_3$ is homogeneous and get $2 \mathcal {L}_+ = 2^{1/3} \textsf{ag}_3\left( 1, 2^{-1/3}\right) = \textsf{ag}_3\left( 2^{1/3}, 1\right) .$

We note that the connection $b(e^{-\frac{2\pi }{\sqrt{3}}}) = 1/(2 \mathcal {L}_+)$ was already established by one of the authors in [29] and we mainly filled in some details. The special value $b(e^{-2\pi /\sqrt{3}})$ also plays a role in the theory of Dixon elliptic functions (see [22]). Also, we want to briefly mention that the second lemniscate constant $\ell _2 = 1/(2\theta _4(e^{-\pi })^2)$ appears in Landau’s problem as well [29] (see [4, 26] for the restricted problems in question). Originally, it appeared as the ratio of the arc length of the lemniscate of Bernoulli to the arc length of the unit circle. The computation of the arc length of the lemniscate involves elliptic integrals and Gauss computed it by hand:

$$\begin{aligned} 2 \varpi = 4 \int _0^{\pi /2} \frac{d x}{\sqrt{2 \left( 1 - \frac{1}{2} \sin (x)^2\right) }} = 5.24412 \ldots \, . \end{aligned}$$

Thus, the ratio of $2 \varpi$ to the arc length of the unit circle is $\frac{2 \varpi }{2 \pi } = 0.834627 \ldots = G.$ Gauss observed that this is numerically $1/\textsf{ag}_2(\sqrt{2},1)$. In fact, he was so enthusiastic about the connection he had found, that on May 30, 1799, he wrote in his diary (translated from [50]):

*We have established that the arithmetic–geometric mean between 1 and *$\sqrt{2}$ *is* $\frac{\pi }{\varpi }$ *to the* $11^{\text {th}}$ *decimal place; the demonstration of this fact will surely open an entirely new field of analysis.*

We refer to the article by Cox [23] for more historical background and the proof Gauss actually found for his observation. Insights into the AGM for complex numbers are also given there.

**Fig. 4:**
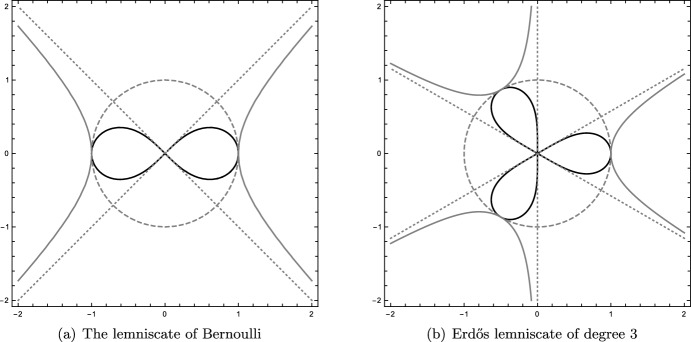
The lemniscate of Bernoulli and the Erdős lemniscate of degree 3 (put to scale). The reflection of the respective lemniscate with respect to the unit circle (inverting distances) yields the corresponding hyperbolas. The intersections of their asymptotes with the unit circle yield root systems. These generate a (scaled and rotated) von Neumann lattice and a hexagonal lattice, respectively

It stands to reason that the constant $\mathcal {L}_+$ will also play a role in the problem of finding the longest polynomial lemniscate of degree 3 (see Fig. 4). For a monic polynomial *p*(*z*) of degree $n \ge 1$, the polynomial lemniscate is $L_p = \{z \in \mathbb {C}\mid |p(z)| = 1\}$ (see [36]).

An open conjecture of Erdős, Herzog, and Piranian [25, Problem 12] states that for any fixed $n \ge 1$, the polynomial $p_0(z) = z^n - 1$ gives the maximal length of the lemniscate $L_p$ among all lemniscates of degree *n*. The lemniscates $L_{p_0}$ are also called Erdős lemniscates.

## Gabor frame bounds and the AGM

Our interest lies in computing Gaussian Gabor frame bounds. The results below show that they obey the AGM machinery and, hence, are intimately connected to the theory of elliptic integrals, hypergeometric functions and analytic number theory. However, this is only a side remark and we will not study these connections in depth here. We remark again that, classically, the frame operator is defined without the normalizing constant $\text {vol}(\Lambda )$. However, we want to define it in this way for two reasons:

- In this way the frame bounds fulfill $A_\Lambda \le 1 \le B_\Lambda$, rather than $A_\Lambda \le \text {vol}(\Lambda )^{-1} \le B_\Lambda$.
- We do not need to re-normalize results, applicable to the frames bounds, which have been obtained by Gauss himself.

### Computing Gabor frame bounds

Determining whether the Gabor frame operator satisfies the spectral inequality (3.3) is a difficult task, at best. There have been several theoretical methods developed to investigate whether a Gabor system is a Gabor frame. In the case of integer or rational density, a popular method is the Zak transform [42, Chap. 8], which was introduced by Zak in the context of solid-state physics [71]. Alternatively, one can turn to duality theory. We present only the most essential results which are needed for our purpose. For more details, we refer to the results of Daubechies, Landau and Landau [24], Janssen [47], Ron and Shen [61] and Wexler and Raz [68]. For a more recent treatise on the topic, we refer to [41].

We have already seen how we can use the relaxed condition (4.2) and FIGA (4.4) to estimate the spectral bounds $A_\Lambda$ and $B_\Lambda$ of the Gabor frame operator $S_\Lambda$. We will now use the mentioned duality theory, which usually involves the (symplectic) Poisson summation formula, to compute exact bounds. By duality, we mean characterizing the Gabor systems $\mathcal {G}(g, \Lambda )$ which satisfy the frame inequality by using a condition on the system $\mathcal {G}(g,\Lambda ^\circ )$. Part of the theory allows us to relate the spectral bounds of the Gabor frame operator to sums over the adjoint lattice. We use a result of Janssen [48] (see also [30] for further applications) in the generalized form given in [27], tailored to our situation and normalization.

#### Proposition 5.1

(Janssen [48]) For the Gaussian window $\varphi (t) = 2^{1/4} e^{-\pi t^2}$ and a lattice $\Lambda = \Lambda (\alpha ) \subseteq \mathbb {R}^2$ of density $\alpha = 2N$, $N \in \mathbb {N}$, consider the Gabor system $\mathcal {G}(\varphi , \Lambda )$. Then the spectral bounds of the associated frame operator $S_\Lambda$ are given by

$$\begin{aligned} \begin{aligned} A_\Lambda&= \min _{z \in \mathbb {R}^2} \sum _{\lambda ^\circ \in \Lambda ^\circ } e^{-\frac{\pi }{2} |\lambda ^\circ |^2} e^{2 \pi i \sigma (\lambda ^\circ ,z)},\\ B_\Lambda&= \max _{z \in \mathbb {R}^2} \sum _{\lambda ^\circ \in \Lambda ^\circ } e^{-\frac{\pi }{2} |\lambda ^\circ |^2} e^{2 \pi i \sigma (\lambda ^\circ ,z)}. \end{aligned} \end{aligned}$$

Above, the skew-symmetric form $\sigma (\lambda ^\circ , z) = -\sigma (z, \lambda ^\circ ) = \lambda ^\circ \cdot \mathcal {J} z$ appears as a consequence of the symplectic structure of the time-frequency plane (see [35, 39]). However, this is merely a rotation of the co-ordinates and we could write the result using the dual lattice $\Lambda ^\perp$ and the Euclidean inner product $\lambda ^\perp \cdot z$ in the complex exponential (see also [27]).

### The proof of Theorem 1.2

#### Proof

For the proof, we use Proposition 5.1. The problem is to find extremizers for

$$\begin{aligned} \widehat{\theta }_{\Lambda _{1\times 1}(1)}(b;\alpha ) = \sum _{k,l \in \mathbb {Z}} e^{-\pi \alpha (k^2+l^2)} e^{2 \pi i (k b_2 - l b_1)}, \quad b=(b_1,b_2) \in \mathbb {R}^2. \end{aligned}$$

Note that computing spectral bounds for lattice density 2*N*, $N \in \mathbb {N}$, corresponds to choosing positive integer values *n* for the parameter $\alpha$ of the above lattice theta function. This discrepancy of the factor 2 in the exponent mainly comes from the fact that

$$\begin{aligned} |\langle \pi (z) \varphi , \pi (\lambda ) \varphi \rangle | = e^{-\frac{\pi }{2} |\lambda -z|^2}. \end{aligned}$$

An application of the triangle inequality shows that the maximum of $\widehat{\theta }_\Lambda (b;\alpha )$ (for any $\Lambda$ and any $\alpha > 0$) is always attained for $b = (0,0)$ and, by periodicity at any other lattice point. Determining the exact value of *b* which gives the minimum is in general a challenging task [12] (see also [3]). Due to the occurring symmetries in the von Neumann lattice, the minimum is always attained in the center of a fundamental square, i.e., at $b \in (1/2,1/2)+\mathbb {Z}^2$ (compare [48] and also [13]) times the right scaling factor. For the scaled von Neumann lattice $\Lambda _{1\times 1}(\alpha ) = {\alpha }^{-1/2} \mathbb {Z}\times {\alpha }^{-1/2} \mathbb {Z}$, $\alpha = 2N$ with $N \in \mathbb {N}$, we recall from Janssen’s article [48] that the explicit Gaussian Gabor frame bounds are obtained as squares of Jacobi theta functions, where we set $q = e^{-\pi }$:

$$\begin{aligned} A_{1\times 1}(2N)&= \sum _{k,l \in \mathbb {Z}} (-1)^{k+l} e^{-\pi N (k^2+l^2)} = \theta _4(q^N)^2,\\ B_{1\times 1}(2N)&= \sum _{k,l \in \mathbb {Z}} e^{-\pi N (k^2+l^2)} = \theta _3(q^N)^2. \end{aligned}$$

Now, if $\alpha$ is a (positive) power of 2, say $\alpha = 2^n$, $n \in \mathbb {N}_+$, then we have Gabor systems over the following scaled family of von Neumann lattices:

$$\begin{aligned} \Lambda _{1\times 1}(2^{n}) = 2^{-n/2} \mathbb {Z}\times 2^{-n/2} \mathbb {Z}. \end{aligned}$$

We obtain the spectral bounds of the Gabor frame operator as

$$\begin{aligned} A_{1\times 1}(2^n) = \theta _4(q^{2^n})^2 \quad \text { and } \quad B_{1\times 1}(2^n) = \theta _3(q^{2^n})^2. \end{aligned}$$

All that is left to observe is that we can now use the arithmetic–geometric mean iteration for Jacobi’s theta functions:

5.1 $$\begin{aligned} \theta _3(q)^2+\theta _4(q)^2 = 2 \theta _3(q^2)^2 \quad \text { and } \quad \theta _3(q) \theta _4(q) = \theta _4(q^2)^2. \end{aligned}$$

This implies that

$$\begin{aligned} B_{1\times 1}(2^{n+1}) = \frac{A_{1\times 1}(2^n)+B_{1\times 1}(2^n)}{2} \quad \text { and } \quad A_{1\times 1}(2^{n+1}) = \sqrt{A_{1\times 1}(2^n) B_{1\times 1}(2^n)}. \end{aligned}$$

$$\square $$

Note that in the (AGM-)formulas in (5.1) the only restriction on the nome *q* is $0<|q|<1$. As the bounds in Proposition 5.1 hold for any even lattice density, we are actually not restricted to start with density 2 in Theorem 1.2. The formulas (1.1) for the bounds in Proposition 1.1 hold for any lattice $\Lambda _{1\times 1}(2N)$, $N \in \mathbb {N}$ by simply setting $q=e^{-N\pi }$ and we can adjust Theorem 1.2 accordingly. The case of odd lattice densities does not seem to lead to simple iterations. Using results in [48] (see also [31]), we have for density $2N-1$, $N \in \mathbb {N}$ and $q=e^{-\pi }$

$$\begin{aligned} A_{1\times 1}(2N-1)&= \theta _4\left( q^{\frac{2N-1}{2}}\right) ^2 - 2 \theta _2\left( q^{2(2N-1)}\right) ^2,\\ B_{1\times 1}(2N-1)&= \theta _3\left( q^{\frac{2N-1}{2}}\right) ^2 - 2 \theta _2\left( q^{2(2N-1)}\right) ^2. \end{aligned}$$

The case of rational densities involves even more complicated algebraic expressions of Jacobi theta functions. The theory to obtain exact frame bounds in that case is available (see, e.g., [42, Chap. 8.3]), but, to the best of our knowledge, the specific formulas have not been worked out so far. The difficulty arises from the non-commutativity of the time-frequency shifts (cf. (3.1)). We are not aware of a method giving exact formulas for irrational densities.

Turning back to our original statement, the spectral bounds for the frame operator for $\Lambda _{1\times 1}(2) = 2^{-1/2} \mathbb {Z}\times 2 ^{-1/2} \mathbb {Z}$ have a simple algebraic dependence: we know that (see [28, 66])

$$\begin{aligned} \kappa _{1\times 1}(2) = \frac{B_{1\times 1}(2)}{A_{1\times 1}(2)} = \sqrt{2} \quad \Longleftrightarrow \quad B_{1\times 1}(2) = \sqrt{2} A_{1\times 1}(2). \end{aligned}$$

This goes back to the Jacobi identity $\theta _2(q)^4+\theta _4(q)^4 = \theta _3(q)^4$. We can now use our knowledge that $A_{1\times 1}(2^n)$ and $B_{1\times 1}(2^n)$ obey the $\textsf{ag}_2$ machinery to iteratively compute condition numbers $\kappa _{1\times 1}(2^n) = B_{1\times 1}(2^n)/A_{1\times 1}(2^n)$ of the associated family of frame operators.

$$\begin{aligned} B_{1\times 1}(2^{n+1}) = (1+\kappa _{1\times 1}(2^n)) \frac{A_{1\times 1}(2^n)}{2} \quad \text { and } \quad A_{1\times 1}(2^{n+1}) = \sqrt{\kappa _{1\times 1}(2^n)} \, A_{1\times 1}(2^n). \end{aligned}$$

It readily follows that the sequence of condition numbers obeys the iterative rule

$$\begin{aligned} \kappa _{1\times 1}(2^{n+1}) = \frac{1}{2} \left( \frac{1}{\sqrt{\kappa _{1\times 1}(2^n)}} + \sqrt{\kappa _{1\times 1}(2^n)} \right) . \end{aligned}$$

As $\kappa _{1\times 1}(2^n) \ge 1$, it is simple to see that the sequence of condition numbers is decreasing:

$$\begin{aligned} \kappa _{1\times 1}(2^n) \ge \sqrt{\kappa _{1\times 1}(2^n)} \ge \frac{1}{2} \left( \frac{1}{\sqrt{\kappa _{1\times 1}(2^n)}} + \sqrt{\kappa _{1\times 1}(2^n)} \right) = \kappa _{1\times 1}(2^{n+1}). \end{aligned}$$

In fact, it is converging to 1. This shows that the frame operator converges to the identity operator as the density tends to infinity. We note that this is only a special case of a result of Feichtinger and Zimmermann [33]. However, the above proof has already been accessible to Gauss and implies the existence of Gabor frames if the density of the lattice is high enough. Obviously, it considerably precedes the theory of Gabor frames, which makes it especially interesting. We will now recall a result of Gauss^1^ [38, Theorem (23), p.467] (see also [50, Entry 102]) formulated in the language of Jacobi theta functions.

#### Theorem

(Gauss, 1799) The arithmetic–geometric mean of $\theta _3(q)^2$ and $\theta _4(q)^2$ always equals 1.

Note that Gauss’ theorem shows that the spectral bounds $A_{1\times 1}(2^n)$ and $B_{1\times 1}(2^n)$ of the frame operator $S_{\Lambda _{1\times 1}}$ both tend to 1 as $n \rightarrow \infty$. Hence, the double-sided operator inequality

$$\begin{aligned} A_n \textsf{I}_{L^2} \le S_{\Lambda _{2^{-n}}} \le B_n \textsf{I}_{L^2} \end{aligned}$$

shows that $S_{\Lambda _{2^{-n}}} \rightarrow \textsf{I}_{L^2}$ in the operator norm. We know that the rate of convergence is quadratic [17], which fits nicely with doubling the lattice density in each iteration of $\textsf{ag}_2$:

$$\begin{aligned} \textsf{ag}_2(B_{1\times 1}(2), A_{1\times 1}(2)) = \textsf{ag}_2(B_{1\times 1}(2^n), A_{1\times 1}(2^n)) = 1. \end{aligned}$$

### The proof of Theorem 1.3

#### Proof

We begin with a short overview of the hexagonal lattice. It can be realized as

$$\begin{aligned} \Lambda _2(\alpha ) = \alpha ^{-1/2} H\mathbb {Z}^2, \quad \text { where } \quad H = \tfrac{\sqrt{2}}{\root 4 \of {3}} \begin{pmatrix} 1 &  \frac{1}{2} \\ 0 &  \frac{\sqrt{3}}{2} \end{pmatrix}, \, \alpha > 0. \end{aligned}$$

The circumcenter of the fundamental triangle with vertices (0, 0), *H*(1, 0) and *H*(0, 1) is easily verifiable to be the point $c_\circ = H(1/3,1/3) = 2^{-1/2}3^{-1/4}(1,\ 3^{-1/2})$. The optimal frame bounds are determined with Janssen’s result [48] again, combined with standard symplectic and metaplectic methods (cf. [27, 28]). As mentioned earlier, for the scaled hexagonal lattice of density 2*N* for some $N\in \mathbb {N}$, it suffices to evaluate at the origin for the upper bound. Evaluating at the circumcenter of a fundamental triangle, for instance, at $\frac{1}{2N} c_\circ$, gives the lower frame due to a result of Baernstein [3]. We obtain explicit Gaussian Gabor frame bounds. Setting $\alpha = 2N$ and $q = e^{-\frac{2 \pi }{\sqrt{3}}}$, we get (cf. Sect.  4.6)

$$\begin{aligned} A_2(\alpha )&= \sum _{k,l \in \mathbb {Z}} e^{2\pi i \frac{(k-l)}{3}} e^{-\frac{2\pi }{\sqrt{3}} N (k^2+kl+l^2)} = b(q^N),\\ B_2(\alpha )&= \sum _{k,l \in \mathbb {Z}} e^{-\frac{2\pi }{\sqrt{3}} N (k^2+kl+l^2)} = a(q^N). \end{aligned}$$

Now, if $\alpha$ is twice a (non-negative) power of 3, say $\alpha = 2\cdot 3^{n-1}$, $n \in \mathbb {N}$, then (similarly to the family of von Neumann lattices) we concern ourselves with the following family of lattices: $\Lambda _2(2\cdot 3^{n-1}) = 2^{-1/2}3^{-(n-1)/2} H\mathbb {Z}^2$. We see that the spectral bounds can be written as

$$\begin{aligned} A_2 (2 \cdot 3^{n-1}) = b(q^{3^{n-1}}) \quad \text { and } \quad B_2 (2 \cdot 3^{n-1}) = a(q^{3^{n-1}}). \end{aligned}$$

As established in Sect. 4.3, (4.1), the sequences $\left( A_2(2\cdot 3^{n-1})\right) _{n\in \mathbb {N}}$ and $\left( B_2(2\cdot 3^{n-1})\right) _{n\in \mathbb {N}}$ comply to the cubic arithmetic–geometric mean construction;

$$\begin{aligned} B_2(2 \cdot 3^n)&= \frac{B_2(2 \cdot 3^{n-1}) + 2 A_2(2 \cdot 3^{n-1})}{3}\\ A_2(2 \cdot 3^n)&= \root 3 \of {A_2(2 \cdot 3^{n-1}) \, \frac{A_2(2 \cdot 3^{n-1})^2 + A_2(2 \cdot 3^{n-1}) B_2(2 \cdot 3^{n-1}) + B_2(2 \cdot 3^{n-1})^2}{3}} \, . \end{aligned}$$

$$\square $$

A simple calculation with the Poisson summation formula relates *b*(*q*) and *c*(*q*) analogously to the famous formula for $$\theta _4(q)$$ and $$\theta _2(q)$$. In particular, it shows $$B_2 (2)^3 = 2 A_2(2)^3 $$. Equivalently, the condition number is $$\kappa _2(2) = \root 3 \of {2}$$. Inductively, we obtain

$$\begin{aligned} \kappa _2(2\cdot 3^{n})^3 =&\frac{1}{9}\cdot \frac{(B_2(2\cdot 3^{n-1})+2A_2(2\cdot 3^{n-1}))^3}{A_2(2\cdot 3^{n-1})(A_2(2\cdot 3^{n-1})^2+A_2(2\cdot 3^{n-1})B_2(2\cdot 3^{n-1})+B_2(2\cdot 3^{n-1})^2)} \\&= \frac{1}{9}\cdot \left( \tfrac{B_2(2\cdot 3^{n-1})}{A_2(2\cdot 3^{n-1})}+2\right) ^3 \left( 1+\tfrac{B_2(2\cdot 3^{n-1})}{A_2(2\cdot 3^{n-1})}+\left( \tfrac{B_2(2\cdot 3^{n-1})}{A_2(2\cdot 3^{n-1})}\right) ^2\right) ^{-1}\\ =&\frac{1}{9}\cdot \left( \kappa _2(2\cdot 3^{n-1})+2\right) ^3 \left( 1+\kappa _2(2\cdot 3^{n-1})+\kappa _2(2\cdot 3^{n-1})^2\right) ^{-1}, \\ \kappa _2(2\cdot 3^{n}) =&\frac{\root 3 \of {3}}{3}\cdot \left( \kappa _2(2\cdot 3^{n-1})+2\right) \left( 1+\kappa _2(2\cdot 3^{n-1})+\kappa _2(2\cdot 3^{n-1})^2\right) ^{-1/3}. \end{aligned}$$

We observe that the sequence of condition numbers is decreasing because

$$\begin{aligned} \Big (\frac{\kappa _2(2\cdot 3^{n})}{\kappa _2(2\cdot 3^{n-1})}\Big )^3 \!=\! \frac{1}{9}\cdot \frac{1+2 \kappa _2(2\cdot 3^{n\!-\!1})^{-1}}{\left( 1\!+\!\kappa _2(2\cdot 3^{n-1})\!+\!\kappa _2(2\cdot 3^{n-\!1})^2\right) ^{1/3}} \!<\! \frac{1}{9}\cdot \frac{1\!+\!\tfrac{2}{1}}{\left( 1\!+\!1+1\right) ^{1/3}} <1. \end{aligned}$$

Independently of the result of Feichtinger and Zimmermann [33], we will conclude that the frame operator tends to the identity. This follows from properties of cubic theta functions.

#### Theorem

(Borwein and Borwein, 1991) The cubic arithmetic–geometric mean of *a*(*q*) and *b*(*q*) always equals 1.

The proof can be found in [17, Thm. 2.3]. As for the von Neumann lattice, this implies the convergence $S_{\Lambda _2(2\cdot 3^{n})} \rightarrow \textsf{I}_{L^2}$, $n \rightarrow \infty$. The rate of convergence is cubic [16, Chap. 1.1], [17], which fits nicely with the triplication of the lattice density in each iteration of $\textsf{ag}_3$:

$$\begin{aligned} \textsf{ag}_3(B_2(2), A_2(2)) = \textsf{ag}_3(B_2(2 \cdot 3^n), A_2(2 \cdot 3^n)) = 1. \end{aligned}$$

Note that similar to the case of the von Neumann lattice, it is not necessary to start the iteration process with the hexagonal lattice of density 2. Any scaled hexagonal lattice of even density allows for the $\textsf{ag}_3$ iteration. The issues for odd and (ir-)rational densities remain.

## Rectangular lattices

We will now present some results for rectangular lattices, as their frame bounds can be expressed by means of the frame bounds for the von Neumann lattice. We will handle lattices of even density. In that case, the optimal frame bounds for a lattice $\Lambda _{a \times b}(\alpha ) = \alpha ^{-1/2} (a \mathbb {Z}\times b \mathbb {Z})$ with $\alpha = 2N$, $N \in \mathbb {N}$, $ab = 1$ and $q = e^{-\pi }$ are given by (see [31])

$$\begin{aligned} A_{a \times b} (2N)&= \theta _4(q^{N a^2})\theta _4(q^{N b^2}),\\ B_{a \times b} (2N)&= \theta _3(q^{Na^2})\theta _3(q^{Nb^2}). \end{aligned}$$

We observe what happens by doubling the density while preserving the initial ratio *a* : *b*.

$$\begin{aligned} A_{a \times b} (2^{n+1})&= \theta _4(q^{2^{n} a^2})\theta _4(q^{2^{n} b^2})\\&= \sqrt{\theta _4(q^{2^{n-1} a^2}) \, \theta _3(q^{2^{n-1} a^2}) \, \theta _4(q^{2^{n-1} b^2}) \, \theta _3(q^{2^{n-1} b^2})}\\&= \sqrt{A_{a \times b} (2^n) \, B_{a \times b} (2^n)} \, . \end{aligned}$$

The situation with the upper bound is a bit more involved.

$$\begin{aligned} B_{a \times b} (2^{n+1})&= \theta _3(q^{2^{n} a^2}) \theta _3(q^{2^{n} b^2})\\&= \left( \frac{\theta _3(q^{2^{n-1} a^2})^2+\theta _4(q^{2^{n-1} a^2})^2}{2} \ \cdot \ \frac{\theta _3(q^{2^{n-1} b^2})^2+\theta _4(q^{2^{n-1} b^2})^2}{2}\right) ^{1/2} \\&= \frac{1}{2}\bigg (\theta _3(q^{2^{n-1} a^2})^2\theta _3(q^{2^{n-1} b^2})^2 + \theta _3(q^{2^{n-1} a^2})^2\theta _4(q^{2^{n-1} b^2})^2 \\&\qquad \qquad + \theta _4(q^{2^{n-1} a^2})^2\theta _3(q^{2^{n-1} b^2})^2 + \theta _4(q^{2^{n-1} a^2})^2\theta _4(q^{2^{n-1} b^2})^2 \bigg )^{1/2}. \end{aligned}$$

As all standard relations between theta functions rely on an integer ratio between *a* and *b*, further meaningful transformations are not readily available. We may simplify the quantities involving theta functions of the same kind:

$$\begin{aligned} \theta _3(q^{2^{n-1} a^2})^2\theta _3(q^{2^{n-1} b^2})^2\! =\! B_{a \times b}(2^n)^2 \quad \! \text { and } \quad \! \theta _4(q^{2^{n-1} a^2})^2\theta _4(q^{2^{n-1} b^2})^2 \!=\! A_{a \times b}(2^n)^2. \end{aligned}$$

However, it is unclear how to treat the terms involving theta functions of different kinds mixed together, as the lattice constants *a* and *b* only need to fulfill $a,b \in \mathbb {R}_+$, $ab = 1$.

## Two conjectures

To our knowledge, the following conjecture has not appeared in a printed form before, but, nonetheless, the authors cannot claim any credit for coming up with this conjecture. It was brought forward to one of the authors in private correspondence with Y. Lyubarskii in 2018 when both were affiliated with NTNU Trondheim. Y. Lyubarskii, on the other hand, was informed about the conjecture by T. Strohmer already in 2009 in a private correspondence. The following conjecture should therefore, to the best of our knowledge, be addressed to T. Strohmer from UC Davis and we publish it with the consent of the originator.

### Conjecture 7.1

(Strohmer, 2009) Let $\kappa _3(\alpha )$ and $\kappa _4(\alpha )$ denote the frame condition number of the Gaussian Gabor system with hexagonal $\Lambda _2(\alpha ) = \alpha ^{-1/2} H \mathbb {Z}^2$ and von Neumann lattice $\Lambda _{1\times 1}(\alpha ) = \alpha ^{-1/2} \mathbb {Z}^2$, respectively, of density $\alpha$. Then, the results in [15] and [44] imply that

$$\begin{aligned} \kappa _3(\alpha ) - \frac{C_3}{1-\frac{1}{\alpha }} \rightarrow 0 \quad \text { and } \quad \kappa _4(\alpha ) - \frac{C_4}{1-\frac{1}{\alpha }} \rightarrow 0, \end{aligned}$$

as we approach the critical density $\alpha \rightarrow 1$ from above. The conjectural exact constants are

$$\begin{aligned} C_r = \frac{2 \pi r}{\tan (\frac{\pi }{r})} \frac{\Gamma (\frac{2}{r})^2}{\Gamma (\frac{1}{r})^4}, \quad r = 3,4. \end{aligned}$$

Plugging in the values, we obtain

$$\begin{aligned} C_3 = \frac{6 \pi }{\sqrt{3}} \, \frac{\Gamma (2/3)^2}{\Gamma (1/3)^4} \approx 0.387438 \ldots \quad \text { and } \quad C_4 = 8 \pi \, \frac{\Gamma (1/2)^2}{\Gamma (1/4)^4} \approx 0.456947 \ldots \, . \end{aligned}$$

This would provide evidence that the conjecture of Strohmer and Beaver raised in [66] holds close to the critical density. At least it shows that asymptotically Gaussian Gabor frames over the hexagonal lattice have smaller condition numbers than over the square lattice:

Assume $\kappa _3(\alpha ) < \kappa _4(\alpha )$ for all $\alpha > 1$ (cf. the conjecture of Strohmer and Beaver in [66]). The asymptotics in Conjecture 7.1, proved in [15] (square lattice) and in [44] (general setting), imply that for every $\varepsilon > 0$ there exists $\alpha _0 > 1$ such that for all $\alpha \in (1, \alpha _0)$ we have

$$\begin{aligned} \left| \kappa _3(\alpha ) - \frac{C_3}{1-\frac{1}{\alpha }}\right|< \varepsilon \quad \text { and } \quad \left| \kappa _4(\alpha ) - \frac{C_4}{1-\frac{1}{\alpha }}\right| < \varepsilon . \end{aligned}$$

Rearranging the inequalities for $\kappa _3(\alpha )$ and $\kappa _4(\alpha )$ yields

$$\begin{aligned} - \varepsilon + \frac{C_3}{1-\frac{1}{\alpha }}< \kappa _3(\alpha )< \varepsilon + \frac{C_3}{1-\frac{1}{\alpha }} \quad \text { and } \quad - \varepsilon + \frac{C_4}{1-\frac{1}{\alpha }}< \kappa _4(\alpha ) < \varepsilon + \frac{C_4}{1-\frac{1}{\alpha }}. \end{aligned}$$

We obtain the following chain of inequalities:

$$\begin{aligned} 0< \kappa _4(\alpha ) - \kappa _3(\alpha ) < \varepsilon + \frac{C_4}{1-\frac{1}{\alpha }} + \varepsilon - \frac{C_3}{1-\frac{1}{\alpha }} = 2 \varepsilon + \frac{C_4 - C_3}{1-\frac{1}{\alpha }}. \end{aligned}$$

As $\varepsilon > 0$ and $1-\frac{1}{\alpha } > 0$, this leads to

$$\begin{aligned} C_4 + 2 \varepsilon (1-\tfrac{1}{\alpha })> C_3 \quad \Longrightarrow \quad C_4 > C_3, \; \text { as } \varepsilon \rightarrow 0 \text { and for } \alpha \rightarrow 1 \text { from above}. \end{aligned}$$

So, we have shown that $C_4$ being larger than $C_3$ is a necessary condition for the conjecture of Strohmer and Beaver [66] (as re-phrased in [2]) to hold. The blow-up of the condition number is not specific to the Gaussian. This asymptotic behavior holds for arbitrary (also non-uniform) Gabor systems with a window in a weighted modulation space as shown in [44].

We boldly formulate another conjecture, based on the conjecture in [66] and the results of Montgomery [56] and Bétermin, Faulhuber, and Steinerberger [12].

### Conjecture 7.2

Let $\kappa _\Lambda (\alpha )$ be the condition number of the frame operator associated to the Gaussian Gabor system $\mathcal {G}(\varphi , \Lambda (\alpha ))$, where $\Lambda (\alpha ) \subset \mathbb {R}^2$ is a lattice of density $\alpha > 1$. Then, there exists a constant $C_\Lambda$, depending on the geometry of the lattice $\Lambda (1)$, such that

$$\begin{aligned} \kappa _\Lambda (\alpha ) - \frac{C_\Lambda }{1-\frac{1}{\alpha }} \rightarrow 0, \end{aligned}$$

as we approach the critical density $\alpha \rightarrow 1$ from above. We conjecture that

7.1 $$\begin{aligned} C_3 \le C_\Lambda , \end{aligned}$$

with equality if and only if $\Lambda$ is the hexagonal lattice.

At the moment, it is not clear to the authors how one should approach Conjecture 7.1 or (7.1) in Conjecture 7.2. One could use a Zak transform approach (or an equivalent approach) computing sequences of condition numbers $\kappa _3(1+1/n)$ and $\kappa _4(1+1/n)$, $n \in \mathbb {N}$. This will become very computationally intensive: one will need to compute and optimize eigenvalues of $n \times n$ matrices where the entries will be lattice theta functions. Maybe there is an approach via modular functions and the theories of Gauss, Ramanujan, and Borwein and Borwein.

## Data Availability

This manuscript has no associated data.

## Footnotes

1 From [38]: *Das Arithmetisch Geometrische Mittel zwischen* $(Px)^2$* und *$(Qx)^2$* ist allemal* $= 1$.
